# Neuropsychiatric symptoms in cognitively normal older persons, and the association with Alzheimer’s and non-Alzheimer’s dementia

**DOI:** 10.1186/s13195-020-00604-7

**Published:** 2020-03-31

**Authors:** Tau Ming Liew

**Affiliations:** 1 Department of Geriatric Psychiatry, Institute of Mental Health, 10 Buangkok View, Singapore, 539747 Singapore; 2 Department of Psychiatry, Singapore General Hospital, Singapore, Singapore; 3 Saw Swee Hock School of Public Health, National University of Singapore, Singapore, Singapore

**Keywords:** Neuropsychiatric symptoms, Psychotic symptoms, Affective symptoms, Agitation, Dementia, Cox regression

## Abstract

**Background:**

Neuropsychiatric symptoms (NPS) have been reported to be useful in predicting incident dementia among cognitively normal older persons. However, the literature has not been conclusive on the differential utilities of the various NPS in predicting the subtypes of dementia. This study compared the risks of Alzheimer’s and non-Alzheimer’s dementia associated with the various NPS, among cognitively normal older persons.

**Methods:**

This cohort study included 12,452 participants from the Alzheimer’s Disease Centers across USA, who were ≥ 60 years and had normal cognition at baseline. Participants completed the Neuropsychiatric Inventory-Questionnaire at baseline and were followed up almost annually for incident dementia (median follow-up = 4.7 years). Symptom clusters of NPS—as identified from exploratory and confirmatory factor-analyses—were included in the Cox regression to investigate their associations with incident dementia.

**Results:**

The various NPS showed independent yet differential associations with incident dementia. Although psychotic symptoms were rarely endorsed by the participants, they predicted much higher risk of dementia (HR 3.6, 95% CI 2.0–6.4) than affective symptoms (HR 1.5, 95% CI 1.2–1.8) or agitation symptoms (HR 1.6, 95% CI 1.3–2.1). Psychotic symptoms predicted all dementia subtypes, while affective and agitation symptoms differentially predicted some subtypes. Across dementia subtypes, psychotic symptoms had relatively higher risk estimates than affective or agitation symptoms, with the risk estimates being particularly high in non-Alzheimer’s dementia.

**Conclusions:**

Among cognitively normal individuals, the presence of NPS may warrant greater clinical vigilance as precursors to dementia and its subtypes. The findings highlight the need for further research to enrich our understanding on the neurobiological links between various NPS and dementia subtypes. They may also change the clinical approach in managing late-life psychotic symptoms, requiring a greater emphasis on dementia surveillance in the diagnostic criteria of late-life psychotic disorders.

## Introduction

In extant literature, there has been much evidence to demonstrate the relevance of neuropsychiatric symptoms (NPS) — especially among individuals with *mild cognitive impairment* [1–5] — in predicting incident dementia. For example, in the population-based Cache County study [3], the presence of NPS, even of mild severity, was associated with conversion from cognitive impairment no dementia to all-cause dementia. In an analysis of the National Alzheimer’s Coordinating Center (NACC) dataset [4], the presence of at least one NPS increased the risk of conversion from mild cognitive impairment to dementia (hazard ratio, HR 1.37; 95% CI 1.1–1.7), while in a separate analysis of the NACC dataset [5], the risk of conversion from mild cognitive impairment to dementia was specifically shown to be associated with affective symptoms (HR 1.6, 95% CI 1.4–1.9) and psychotic symptoms (HR 1.6, 95% CI 1.2–2.2).

Parallel to the literature on NPS in *mild cognitive impairment*, there is also growing evidence to highlight similar relevance of NPS among *cognitively normal* older persons. For example, depression and anxiety have been shown in recent meta-analyses to be risk factors of Alzheimer’s and vascular dementia [6, 7], while early manifestations of disinhibition and apathy are integral to the diagnosis of behavioral variant frontotemporal dementia [8], and hallucinations and delusions (even in the absence of cognitive symptoms) are among the key diagnostic criteria for dementia with Lewy Bodies [9]. These growing evidences have led to the recent inclusion of NPS in the 2018 NIA-AA (National Institute on Aging-Alzheimer’s Association) research framework for Alzheimer’s disease [10], as being part of the transition phase that straddles between the equivalence of normal cognition and mild cognitive impairment.

Yet, several gaps remain in the literature on NPS in *cognitively normal* older persons. NPS comprise heterogeneous clusters of symptoms, such as those related to affective regulation, impulse control, and psychosis [2]. These various NPS have not been *directly compared* among each other, either in their risks of all-cause dementia or across the different subtypes of dementia. As such, the literature has not been certain on the *differential utilities* of the various NPS in predicting dementia or its respective subtypes. Moreover, even though the Neuropsychiatric Inventory-Questionnaire (NPI-Q) [11] is the most widely used screening tool for NPS in older persons [12, 13], its utility has mostly been established among those with *known cognitive impairment* (that is, in mild cognitive impairment and in the various stages of dementia) [13]. It is not yet conclusive that NPI-Q also has similar utility in *cognitively normal* individuals, especially to identify NPS which can be predictive of subsequent dementia. This study sought to examine the comparative utilities of the various NPS in cognitively normal older persons—as identified by NPI-Q—in predicting the subsequent development of Alzheimer’s and non-Alzheimer’s dementia. As a secondary aim, in the subset of participants with complete data on the neuropsychological tests, this study also examined the comparative utilities of the various NPS in predicting subsequent changes in the *Z*-scores of neuropsychological tests.

## Methods

### Participants and procedures

This cohort study is based on the NACC database [14], involving participants who were recruited from approximately 39 Alzheimer’s Disease Centers across the USA between September 2005 and May 2018. The study included participants who fulfilled the following criteria: (1) age ≥ 60 years, (2) diagnosed as having normal cognition at baseline (that is, healthy volunteers who have received clinical evaluations at baseline and found not to have mild cognitive impairment or dementia), (3) no known diagnosis or treatment of schizophrenia, and (4) provided baseline information on NPI-Q. At baseline and on an approximately annual basis, the participants would be accompanied by an informant (who knew the participants well, usually a spouse, child, or friend) and took part in standardized assessments (which included clinical history, physical examination, and detailed neuropsychological testing) to evaluate for incident dementia. All contributing Alzheimer’s Disease Centers obtained informed consent from their participants, as well as received approval by their local institutional review boards.

### Measures

The Neuropsychiatric Inventory-Questionnaire (NPI-Q) [11] was measured at the baseline of this study. NPI-Q is an informant-based questionnaire that screens for the presence of NPS in the *past month* (of note, the focus on the *past month* was emphasized with underscore and boldface in the questionnaire). To avoid capturing NPS that are unrelated to preclinical neurocognitive disorders (such as those related to longstanding psychiatric disorders), the NPI-Q in NACC included the following instructions to the NPI-Q interviewers: “For subjects who are cognitively normal or whose cognition has not yet been evaluated, please report any behaviors or symptoms that were present within the *last month*, ignoring behaviors and symptoms that are usual for the subject and have been customary throughout his/her life”. NPI-Q has 12 items that assess NPS in 12 domains, namely depression, anxiety, apathy, sleep, appetite, agitation, irritability, disinhibition, elation, motor disturbance, delusions, and hallucinations. It was administered to informants by trained healthcare professionals, with each of its items rated on a 4-point Likert scale: 0 = not present, 1 = mild (noticeable, but not a significant change), 2 = moderate (significant, but not a dramatic change), and 3 = severe (very marked or prominent, a dramatic change). To ensure the NPI-Q was administered correctly, all interviewers at Alzheimer’s Research Centers had to undergo a compulsory online certification course (https://www.alz.washington.edu/npiq/Signin2.html) before they were allowed to administer NPI-Q. In the current dataset, the NPI-Q had demonstrated adequate internal-consistency reliability, with a Cronbach’s alpha of 0.74 (95% CI 0.72–0.76).

The diagnoses of mild cognitive impairment and dementia were made based on all available information from standardized assessments [14], with 69.9% made via consensus conference and the remainder made by single clinicians. Mild cognitive impairment was diagnosed using the modified Petersen criteria [15]. Dementia was diagnosed using either the McKhann (1984) criteria [16], DSM-IV (Diagnostic and Statistical Manual of Mental Disorders–Fourth Edition) criteria [17], or McKhann (2011) criteria [18], with further classification into the subtypes of Alzheimer’s dementia [16, 18], vascular dementia [19], dementia with Lewy Bodies [9, 20, 21], frontotemporal lobar degeneration [8, 20, 22–26], and other subtypes.

In NACC database, the detailed neuropsychological battery included cognitive tests [27, 28] which covered the domains of immediate memory (*Craft Story 21 Immediate Recall*), visuospatial abilities (*Benson Complex Figure Copy*), delayed memory (*Craft Story 21 Delayed Recall* and *Benson Complex Figure Recall*), language (*Multilingual Naming Test*, and *Verbal Fluency–Animal and L-words*), attention (*Number Span Test Forward and Backward*), processing speed (*Trail Making Test Part A*), and executive function (*Trail Making Test Part B*). These cognitive tests are briefly described here, with the full details available in a recent publication [27]. *Craft Story 21* assesses the ability to provide verbatim recall of a short story immediately after hearing it, and 20 min later [29]. *Benson Complex Figure Test* assesses the ability to copy a simplified form of the Rey-Osterrieth figure and to draw from memory the same figure after 20 min [30]. *Multilingual Naming Test* assesses the ability to name 32 objects which are shown in picture forms [31]. *Verbal Fluency Test* measures the number of animals and L-words that a participant can name in 1 min. *Number Span Test* assesses the ability to repeat a series of numbers (ranging from 2 to 9 digits), both in the forward and backward sequence. *Trail Making Test* (*Part A*) requires the participants to connect the circles in ascending numerical order (from 1 to 25) as quickly as possible, while *Trail Making Test* (*Part B*) requires the participants to connect the circles while alternating between numbers and letters in an ascending order. Both *Trail Making Tests* (*Part A* and *Part B*) were measured by the correct lines connected divided by the time to completion, as this computation was previously shown to provide more accurate scores [27]. The *Z*-scores for the cognitive tests were computed using the age-, sex-, and education-adjusted normative calculator that was published for the NACC participants [27], while the global *Z*-score was computed by averaging the *Z*-scores of the cognitive tests within the neuropsychological battery.

### Statistical analyses

The factor structure of NPI-Q was first examined to understand how the different NPS cluster with each other in the population of *cognitively normal* older persons. The clustering of the symptoms (instead of the focus on individual symptoms) is useful because most of the NPS tend to co-occur in clinical practice, and hence, this approach can provide more meaningful interpretations of the NPS in clinical practice [12, 13] and has been the suggested approach by some of the researchers in the field [12, 32]. The clustering of symptoms also helps to avoid the issue of multicollinearity among correlated NPS (highly correlated covariates tend to render the results erratic in regression analyses), while at the same time increases the power of NPS in predicting specific subtypes of dementia (rarer symptoms, when evaluated in isolation, tend to have limited power in predicting the less common subtypes of dementia; in contrast, the clustering of NPS increases the number of participants who endorse each symptom cluster and hence improves the overall power of NPS).

To identify the symptom clusters of NPS, the study sample was randomly split into two (based on an 80:20 split), with 80% of the sample used for exploratory factor analysis (EFA) to identify the factor structure of NPI-Q, while the remaining 20% of the sample reserved for confirmatory factor analysis (CFA) to validate the identified factor structure from EFA. This combined approach of EFA and CFA was used because the factor structure of NPI-Q has not been known among the older persons with *normal cognition*. Although the factor structure of NPI-Q has been much studied in the literature [13], the prior factor structures of NPI-Q have been derived from patients with *known cognitive impairment* (that is, mild cognitive impairment and dementia) and may not be directly applied to the current population of older persons with *normal cognition*—this is especially pertinent given the recent suggestion that the factor structure of NPI-Q may vary across different cognitive status (such as normal cognition, mild cognitive impairment, or dementia) [13].

EFA was conducted with maximum likelihood estimation methods and oblique rotation (oblimin), with the number of factors in EFA identified using Horn’s parallel analysis [33]. An item in NPI-Q is deemed to belong to a particular factor if it has a factor loading of ≥ 0.40 in that factor. CFA was conducted in structural equation modeling using maximum likelihood estimation with robust statistics. In CFA, alternative factor structures were also compared to identify the best-fitting model (the other alternative factor structures are further described in the footnote of Additional file 3). The model that fulfilled the criteria of excellent fit (that is, fulfilling all of the following four criteria: Root Mean Square Error of Approximation ≤ 0.05, Standardized Root Mean Square Residual ≤ 0.05, Comparative Fit Index ≥ 0.95, and Tucker-Lewis Index ≥ 0.95) [34] was used to constitute the symptom clusters of NPS in the subsequent analyses.

The symptom clusters of NPS (at baseline) were then included in the Cox proportional-hazard regression—based on the full sample—to evaluate their associations with incident dementia. Time to event was defined as the duration from the baseline visit to the clinical diagnosis of dementia, as well as to the various subtypes of dementia (namely, Alzheimer’s dementia, vascular dementia, dementia with Lewy Bodies, frontotemporal lobar degeneration, and other subtypes of dementia). All the symptom clusters were concurrently included in the Cox regression (that is, each symptom cluster was included as a separate variable) to evaluate the independent risks that were attributable to each of the symptom clusters. They were included as binary variables based on whether the participants endorsed the presence of each symptom cluster at baseline (that is, a symptom cluster would be coded as “yes” when the participants endorsed at least one of the symptoms within the cluster). The Cox regression adjusted for demographic information (age, sex, ethnicity, and years of education) as well as baseline confounders that may potentially predict both the exposure of interest (NPS) and the outcome of interest (dementia) [35], namely, APOE e4 status and use of antidepressants. APOE e4 status was included as a potential confounder because it is a known predictor of dementia (outcome of interest) [36], while at the same time, there has also been reports of its association with NPS (exposure of interest) [37]. Similarly, antidepressant has been reported to be associated with dementia (outcome of interest) [38, 39], and its use may potentially also modify the manifestation of NPS (exposure of interest). The primary analysis was based on participants with follow-up data beyond the baseline visits (also known as complete case analysis). The proportional-hazard assumption of Cox regression was tested statistically based on whether the Schoenfeld residuals were associated with time—in the event there was significant violation of the proportional-hazard assumption (*p* ≤ 0.05 in the global test on statistical significance of non-proportionality), the variables that violated the proportional-hazard assumption were identified using the scaled Schoenfeld residuals and included in the Cox regression as stratified variables [40].

Three sensitivity analyses were conducted to evaluate the consistency of the results when some parts of the Cox regression were modified. They include the following:

1. Using the *severity scores* of each symptom cluster (instead of the binary variables which were based on the presence or absence of each symptom cluster). As described earlier, the items in NPI-Q were scored on 4-point Likert scales. The scores of the respective items in each symptom cluster could be summed to produce the severity score of each symptom cluster.
2. Using inverse probability weighting (IPW) [41] in the Cox regression to account for participants who *did not have follow-up data* (beyond the baseline visits). IPW is a well-accepted strategy to minimize potential bias in the results related to differential risks between those with and without follow-up data. The probabilities of being “complete cases” (those with follow-up data) were generated from logistic regression. The inverse of the probabilities were then used as weights in the Cox regression, so that the results bear more semblance to those who dropped out and are less biased towards participants who provided follow-up data [41, 42]. Further details on IPW are available in Additional file 1.
3. Redefining the symptom clusters by items with *factor loadings of* ≥ *0.20* in the EFA (instead of ≥ 0.40).

In addition, in the subset of participants with complete data on neuropsychological tests, a secondary analysis was conducted to examine the comparative utilities of the symptom clusters of NPS in predicting changes in the *Z*-scores of neuropsychological tests over time. The mixed-effect linear regression was used to account for intra-individual correlations in the repeated measurements of neuropsychological tests. It was conducted assuming unstructured covariance, the random effects of intercept and time (to allow each participant to vary in his neuropsychological scores over time), and the fixed effects of the other covariates in the model. All the statistical analyses were conducted in Stata (version 14).

## Results

**Fig. 1:**
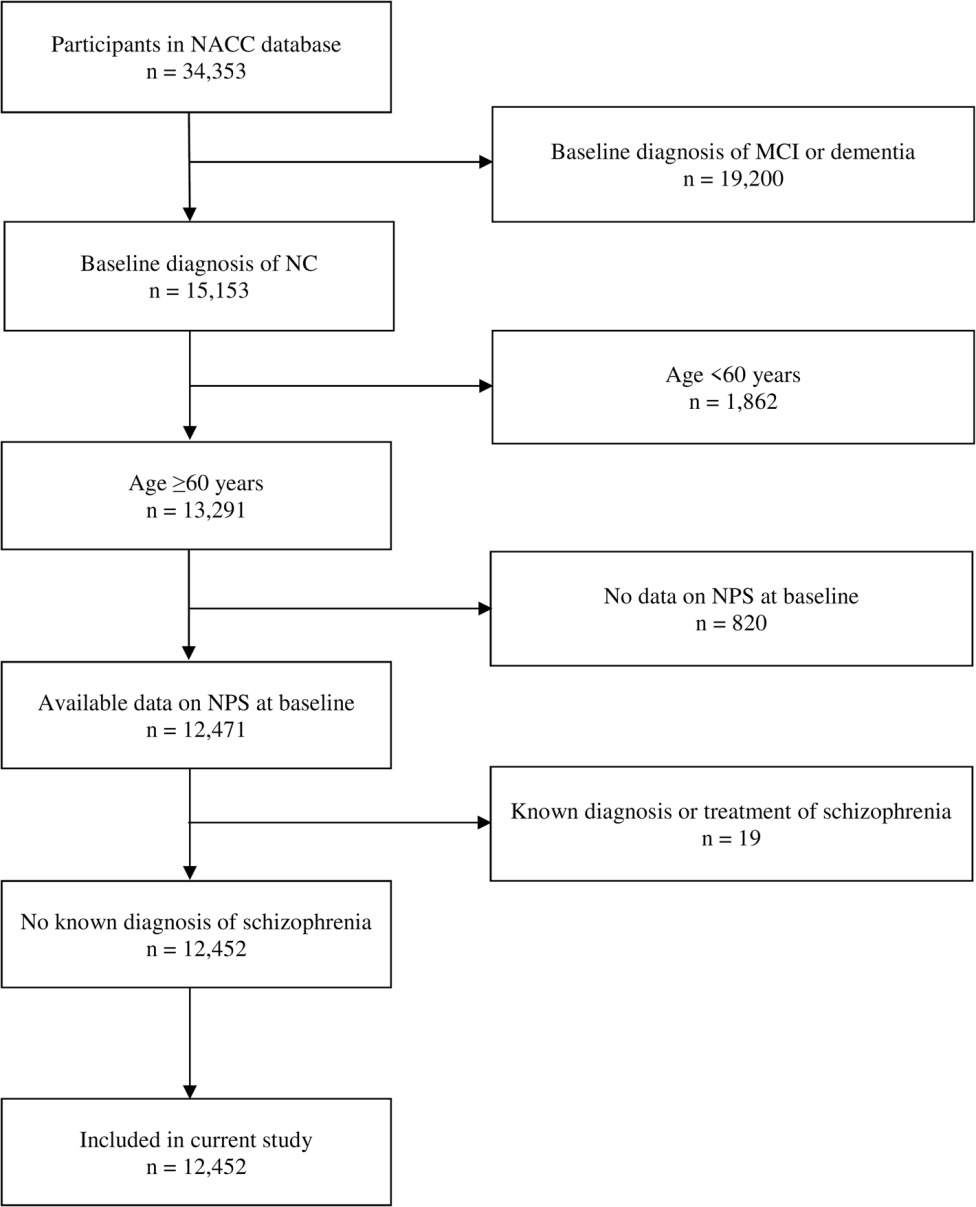
Participant enrolment and exclusion details. MCI, mild cognitive impairment; NACC, National Alzheimer’s Coordinating Center; NC, normal cognition; NPS, neuropsychiatric symptoms

The total sample size was 12,452, with a median age of 72 (interquartile range (IQR) 67–79; full range 60–104) and a median education of 16 years (IQR 14–18; full range 0–29). Figure 1 presents the flow diagram related to participant selection, while Table 1 shows the participant characteristics, as well as the comparison between participants who did and did not develop dementia. About a quarter of the participants (23.0%) only had baseline data and did not contribute to the follow-up data, while the rest of the participants had a median follow-up of 4.7 years (IQR 2.4–7.6 years; full range 0.6–12.4 years)—the comparison of characteristics between those with and without follow-up data is further presented in Additional file 2. At baseline, 31.6% of the participants reported at least one NPS, of which the most common symptoms were related to depression (13.1%), irritability (11.3%), and sleep (10.5%). During follow-up, 724 participants converted to dementia (of which 76.7% were Alzheimer’s dementia, 7.9% vascular dementia, 5.4% dementia with Lewy Bodies, 1.9% frontotemporal lobar degeneration, and 8.2% dementia due other or unknown etiologies).

**Table 1:** Demographic information of the study participants at baseline (*n* = 12,452), and comparison between those did and did not develop dementia during the follow-up period

Variable	Overall sample (*n* = 12,452)	Participants who did not develop dementia (*n* = 11,728)	Participants who developed dementia (*n* = 724)	*p* value^a^
Age, median (IQR; full range)	72 (67–79; 60–104)	72 (67–78; 60–104)	79 (74–84; 60–99)	**< 0.001**
Male sex, *n* (%)	4514 (36.3)	4241 (36.2)	273 (37.7)	0.400
Ethnicity, *n* (%)				**< 0.001**
White	9902 (79.5)	9279 (79.1)	623 (86.0)	
African American	1773 (14.2)	1696 (14.5)	77 (10.6)	
Others/unknown	777 (6.2)	753 (6.4)	24 (3.3)	
Years of education, median (IQR; full range)	16 (14–18; 0–29)	16 (14–18; 0–29)	16 (12–18; 3–29)	**< 0.001**
APOE e4 genotype, *n* (%)				**< 0.001**
Two copies of e4 allele	242 (1.9)	212 (1.8)	30 (4.1)	
One copy of e4 allele	2528 (20.3)	2309 (19.7)	219 (30.2)	
No e4 allele/unknown	9682 (77.8)	9207 (78.5)	475 (65.6)	
History of psychiatric diagnosis, *n* (%)	3513 (28.2)	3308 (28.2)	205 (28.3)	0.950
Use of antidepressants, *n* (%)	2278 (18.3)	2133 (18.2)	145 (20.0)	0.210
Use of anxiolytics, *n* (%)	1436 (11.5)	1362 (11.6)	74 (10.2)	0.260
Use of antipsychotics, *n* (%)	86 (0.7)	83 (0.7)	3 (0.4)	0.350
Presence of NPS, *n* (%)				
Any NPS	3941 (31.6)	3635 (31.0)	306 (42.3)	**< 0.001**
Depression	1629 (13.1)	1488 (12.7)	141 (19.5)	**< 0.001**
Anxiety	1107 (8.9)	1027 (8.8)	80 (11.0)	**0.035**
Apathy	574 (4.6)	506 (4.3)	68 (9.4)	**< 0.001**
Sleep	1306 (10.5)	1212 (10.3)	94 (13.0)	**0.024**
Appetite	686 (5.5)	601 (5.1)	85 (11.7)	**< 0.001**
Agitation	733 (5.9)	658 (5.6)	75 (10.4)	**< 0.001**
Irritability	1411 (11.3)	1301 (11.1)	110 (15.2)	**< 0.001**
Disinhibition	327 (2.6)	293 (2.5)	34 (4.7)	**< 0.001**
Elation	114 (0.9)	99 (0.8)	15 (2.1)	**< 0.001**
Motor disturbance	160 (1.3)	143 (1.2)	17 (2.3)	**0.009**
Delusions	97 (0.8)	78 (0.7)	19 (2.6)	**< 0.001**
Hallucinations	41 (0.3)	35 (0.3)	6 (0.8)	**0.016**

*IQR* interquartile range, *MMSE* Mini-Mental State Examination, *NPS* neuropsychiatric symptoms

^a^Test of difference between participants who did and did not developed dementia during the follow-up period: chi-square test for categorical variables, and the Mann-Whitney *U* test for continuous variables. Boldfaced *p* values are ≤ 0.05

In EFA (based on 80% of the randomly split sample, *n* = 9962), NPI-Q demonstrated three factors among its scale items. The scree plot is shown in Additional file 3, while the results on the three factors are presented in Table 2. Factor 1 comprises *depression*, *anxiety*, and *apathy* and likely represents those with affective symptoms. Factor 2 includes *disinhibition*, *agitation*, and *irritability* and possibly describes NPS related to agitation symptoms. Factor 3 includes *hallucinations* and *delusions* and represents those with psychotic symptoms. Based on pre-specified factor loading of ≥ 0.40, four items from the original NPI-Q (*sleep*, *appetite*, *elation*, and *motor disturbance*) did not load in any of the three factors; however, *sleep* and *appetite* marginally loaded in factor 1 while *elation* and *motor disturbance* marginally loaded in factor 2 (each with factor loading between 0.20 and 0.40). In CFA (based on 20% of the randomly split sample, *n* = 2490), the three-factor model above (based on factor loading of ≥ 0.40 in EFA) was the only one that fulfilled the criteria of excellent fit (Additional file 4). In contrast, the other models that included the four additional items (*sleep*, *appetite*, *elation*, and *motor disturbance*) demonstrated inadequate fit.

**Table 2:** Exploratory factor analysis of the Neuropsychiatric Inventory-Questionnaire and factor loadings of the scale items, based on 80% of the randomly split sample (*n* = 9962). Factor loadings of ≥ 0.40 are highlighted in bold, indicating items which belong to the respective factor. For the purpose of clarity, only factor loadings of ≥ 0.20 are shown in the table

Items in the Neuropsychiatric Inventory-Questionnaire	Factors		
	1	2	3
1. **Depression**—Does the patient seem sad or say that he/she is depressed?	**0.739**		
2. **Anxiety**—Does the patient become upset when separated from you? Does he/she have any other signs of nervousness such as shortness of breath, sighing, being unable to relax, or feeling excessively tense?	**0.515**		
3. **Apathy**—Does the patient seem less interested in his/her usual activities or in the activities and plans of others?	**0.427**		
4. **Sleep**—Does the patient awaken you during the night, rise too early in the morning, or take excessive naps during the day?	0.388		
5. **Appetite**—Has the patient loss or gained weight, or had a change in the type of food he/she likes?	0.237		
6. **Disinhibition**—Does the patient seem to act impulsively, for example, talking to strangers as if he/she knows them, or saying things that may hurt people’s feelings?		**0.654**	
7. **Agitation**—Is the patient resistive to help from others at times, or hard to handle?		**0.652**	
8. **Irritability**—Is the patient impatient and cranky? Does he/she have difficulty coping with delays or waiting for planned activities?		**0.571**	
9. **Elation**—Does the patient appear to feel too good or act excessively happy?		0.368	
10. **Motor disturbance**—Does the patient engage in repetitive activities such as pacing around the house, handling buttons, wrapping string, or doing other things repeatedly?		0.268	
11. **Hallucinations**—Does the patient have hallucinations such as false visions or voices? Does he or she seem to hear or see things that are not present?			**0.562**
12. **Delusions**—Does the patient have false beliefs, such as thinking that others are stealing from him/her or planning to harm him/her in some way?			**0.490**

**Fig. 2:**
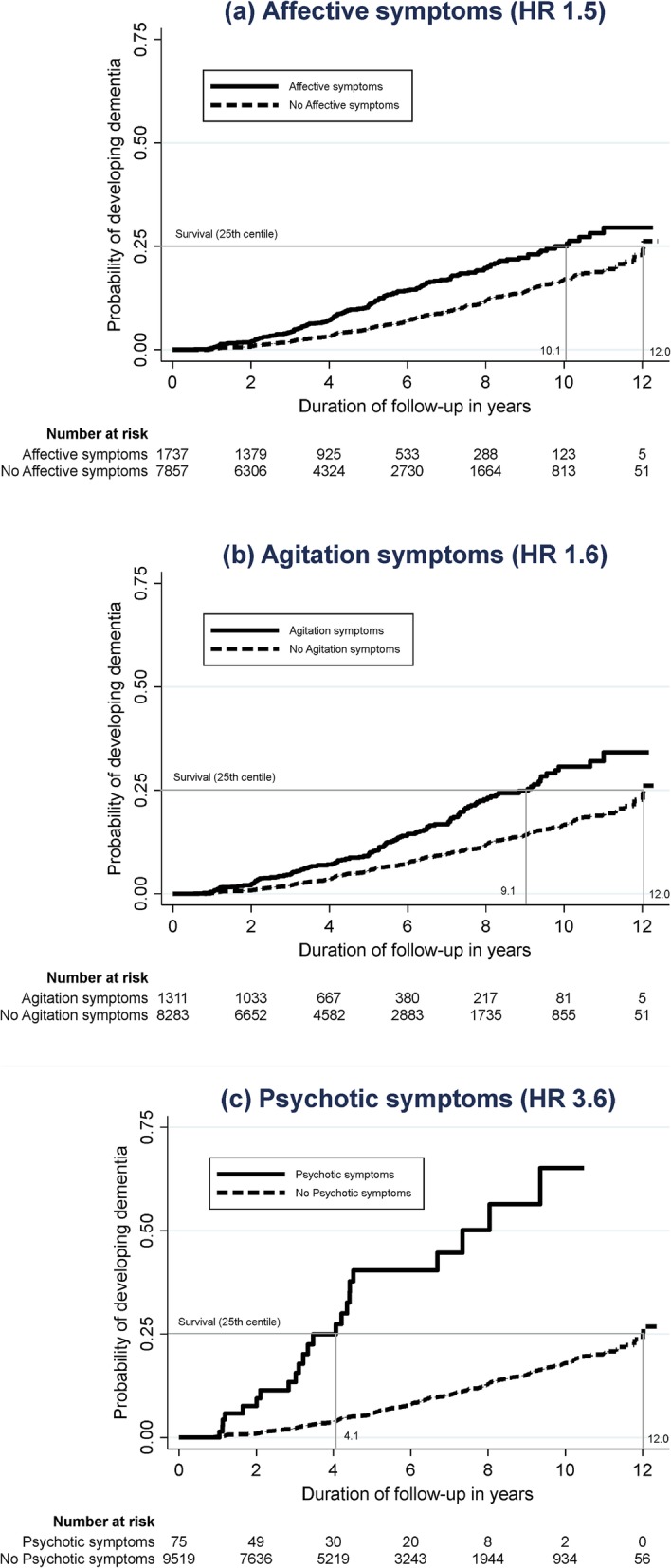
The Kaplan-Meier curves reflecting the risk of dementia in the presence of **a** affective symptoms, **b** agitation symptoms, and **c** psychotic symptoms

Participants who endorsed the three symptom clusters tended to have a higher proportion of incident dementia during the follow-up period. As shown in Table 3, 8.1% of the participants with affective symptoms developed dementia, compared to 5.3% among those without affective symptoms; 8.6% among participants with agitation symptoms compared to 5.4% among those without; and 18.7% among participants with psychotic symptoms compared to 5.7% among those without. The risk associated with the three symptom clusters is further quantified in the Cox regression, with the results presented in Table 4. The three symptom clusters of NPS were significantly associated with the risk of all-cause dementia. However, the risk associated with psychotic symptoms (HR 3.6) was comparatively higher than those of affective (HR 1.5) or agitation symptoms (HR 1.6). This differential risks across the NPS are also visible in the Kaplan-Meier curves in Fig. 2. Among the participants without NPS, a quarter of them would have developed dementia by 12.0 years. This duration shortened to 10.1 years in the presence of affective symptoms, 9.1 years in the presence of agitation symptoms, and 4.1 years in the presence of psychotic symptoms.

**Table 3:** A breakdown of the eventual diagnosis among participants who reported each of the three symptom clusters of neuropsychiatric symptoms at baseline

Dementia subtype	Presence of affective symptoms^a^		Presence of agitation symptoms^a^		Presence of psychotic symptoms^a^	
	Yes (*n* = 2372)	No (*n* = 10,080)	Yes (*n* = 1773)	No (*n* = 10,679)	Yes (*n* = 123)	No (*n* = 12,329)
All-cause dementia	193 (8.1%)	531 (5.3%)	153 (8.6%)	571 (5.4%)	23 (18.7%)	701 (5.7%)
Alzheimer’s dementia	136 (5.7%)	419 (4.2%)	111 (6.3%)	444 (4.2%)	12 (9.8%)	543 (4.4%)
Vascular dementia	15 (0.6%)	42 (0.4%)	9 (0.5%)	48 (0.5%)	2 (1.6%)	55 (0.5%)
Dementia with Lewy Bodies	16 (0.7%)	23 (0.2%)	8 (0.5%)	31 (0.3%)	4 (3.3%)	35 (0.3%)
Frontotemporal lobar degeneration	8 (0.3%)	6 (0.1%)	8 (0.5%)	6 (0.1%)	2 (1.6%)	12 (0.1%)
Other or unknown subtype of dementia	18 (0.8%)	41 (0.4%)	17 (1.0%)	42 (0.4%)	3 (2.4%)	56 (0.5%)

^a^Affective symptoms included depression, anxiety, and apathy. Agitation symptoms included disinhibition, agitation, and irritability. Psychotic symptoms included delusions and hallucinations

**Table 4:** Associations between the neuropsychiatric symptoms (affective, agitation, and psychotic symptoms) and incident dementia

Dementia subtype	Presence of affective symptoms^a^		Presence of agitation symptoms^a^		Presence of psychotic symptoms^a^	
	HR (95% CI)^b^	*p* value	HR (95% CI)^b^	*p* value	HR (95% CI)^b^	*p* value
All-cause dementia	**1.5 (1.2–1.8)**	**< 0.001**	**1.6 (1.3–2.1)**	**< 0.001**	**3.6 (2.0–6.4)**	**< 0.001**
Alzheimer’s dementia	**1.4 (1.1–1.7)**	**0.018**	**1.7 (1.3–2.2)**	**< 0.001**	**2.2 (1.1–4.6)**	**0.027**
Vascular dementia	**1.9 (1.0–3.5)**	**0.042**	1.2 (0.6–2.5)	0.649	**5.7 (1.3–25.6)**	**0.023**
Dementia with Lewy Bodies	**2.8 (1.5–5.3)**	**0.002**	0.7 (0.3–1.7)	0.399	**13.9 (3.8–50.7)**	**< 0.001**
Frontotemporal lobar degeneration	2.7 (0.8–9.4)	0.114	**4.4 (1.3–15.0)**	**0.019**	**8.7 (2.0–38.7)**	**0.004**
Other or unknown subtypes of dementia	1.3 (0.7–2.6)	0.433	**2.2 (1.1–4.3)**	**0.024**	**4.5 (1.4–14.4)**	**0.011**

*HR* hazard ratio, *CI* confidence interval

^a^Affective symptoms included depression, anxiety, and apathy. Agitation symptoms included disinhibition, agitation, and irritability. Psychotic symptoms included delusions and hallucinations

^b^Model adjusted for baseline variables of age, sex, ethnicity, years of education, APOE e4 status, and use of antidepressants. Significant risk estimates (with *p* ≤ 0.05) are highlighted in bold

The three symptom clusters were also examined in their associations with the various subtypes of dementia, as shown in Table 4. Affective symptoms were associated with the risk of Alzheimer’s dementia, vascular dementia, and dementia with Lewy Bodies (HR 1.4–2.8); agitation symptoms were associated with Alzheimer’s dementia, frontotemporal lobar degeneration, and other subtypes of dementia (HR 1.7–4.4), while psychotic symptoms were associated with all the subtypes of dementia (HR 2.2–13.9). Notably, the risk estimates of psychotic symptoms were relatively higher compared to those of the other two symptom clusters, as well as higher in non-Alzheimer’s dementia (HR 4.5–13.9) compared to Alzheimer’s dementia (HR 2.2). The findings remained robust in the three sensitivity analyses, with the results presented in Additional files 5, 6, and 7. In particular, the findings remained similar even when the three symptom clusters were defined by items with factor loadings of ≥ 0.20 in the initial EFA (Additional file 7), which included the additional items of *sleep* and *appetite* in affective symptoms, as well as the additional items of *elation* and *motor disturbance* in agitation symptoms.

In the secondary analysis, the symptom clusters of NPS were further compared in their utilities in predicting changes in the *Z*-scores of neuropsychological tests over time (Table 5). Overall, the 3 symptom clusters were all associated with declines in global *Z*-scores of neuropsychological tests, although psychotic symptoms predicted a larger magnitude of decline in the global *Z*-score (regression coefficient − 0.25) than affective or agitation symptoms (regression coefficient − 0.08, respectively). Affective symptoms affected the visuospatial, memory, processing speed, and executive function domains, and largely spared the language and attention domains. Agitation symptoms affected the language, attention, and executive function domains, and largely spared the visuospatial, memory, and processing speed domains. Psychotic symptoms mainly affected the processing speed and executive function domains, and partially affected the memory and language domains.

**Table 5:** Associations between the neuropsychiatric symptoms (affective, agitation, and psychotic symptoms) and the longitudinal changes in the *Z*-scores of the neuropsychological tests

Neuropsychological tests (*Z*-score)	Cognitive domains	Sample size	Presence of affective symptoms^a^		Presence of agitation symptoms^a^		Presence of psychotic symptoms^a^	
			Regression coefficient (95% CI)^b^	*p* value	Regression coefficient (95% CI)^b^	*p* value	Regression coefficient (95% CI)^b^	*p* value
Global *Z*-score	Global	5380	**− 0.08 (− 0.12, − 0.04)**	**< 0.001**	**− 0.08 (− 0.12, − 0.03)**	**0.001**	**− 0.25 (− 0.44, − 0.06)**	**0.010**
Benson Complex Figure Copy	Visuospatial	6304	**− 0.08 (− 0.15, – 0.01)**	**0.030**	− 0.07 (− 0.15, 0.01)	0.069	− 0.19 (− 0.67, 0.29)	0.431
Craft Story 21 Immediate Recall^c^	Immediate memory	5520	**− 0.14 (− 0.22, − 0.06)**	**< 0.001**	− 0.02 (− 0.11, 0.06)	0.608	− 0.33 (− 0.75, 0.09)	0.121
Craft Story 21 Delayed Recall^c^	Delayed memory	5517	**− 0.18 (− 0.26, − 0.10)**	**< 0.001**	− 0.05 (− 0.14, 0.04)	0.284	**− 0.52 (− 0.86, − 0.18)**	**0.003**
Benson Complex Figure Recall	Delayed memory	6289	**− 0.10 (− 0.17, − 0.03)**	**0.006**	**− 0.10 (− 0.19, − 0.02)**	**0.017**	− 0.27 (− 0.58, 0.04)	0.092
Multilingual Naming Test	Language	5501	− 0.07 (− 0.16, 0.02)	0.106	**− 0.10 (− 0.20, − 0.00)**	**0.041**	**− 0.73 (− 1.17, − 0.28)**	**0.001**
Verbal Fluency–Animal	Language	12,418	**− 0.09 (− 0.13, − 0.05)**	**< 0.001**	**− 0.06 (− 0.11, − 0.02)**	**0.007**	− 0.10 (− 0.28, 0.08)	0.264
Verbal Fluency–L-words	Language	6298	0.00 (− 0.06, 0.07)	0.938	**− 0.11 (− 0.18, − 0.03)**	**0.004**	− 0.13 (− 0.42, 0.15)	0.368
Number Span Test Forward	Attention	5532	− 0.04 (− 0.11, 0.03)	0.253	**− 0.10 (− 0.18, − 0.02)**	**0.014**	− 0.20 (− 0.52, 0.12)	0.217
Number Span Test Backward	Attention	5531	− 0.04 (− 0.11, 0.03)	0.270	**− 0.13 (− 0.21, − 0.05)**	**0.001**	− 0.16 (− 0.50, 0.18)	0.350
Trail Making Test Part A	Processing speed	11,327	**− 0.11 (− 0.15, – 0.07)**	**< 0.001**	− 0.04 (− 0.09, 0.00)	0.061	**− 0.30 (− 0.46, − 0.14)**	**< 0.001**
Trail Making Test Part B	Executive function	11,249	**− 0.11 (− 0.15, − 0.07)**	**< 0.001**	**− 0.07 (− 0.12, − 0.03)**	**0.001**	**− 0.29 (− 0.45, − 0.14)**	**< 0.001**

*CI* confidence interval

^a^Affective symptoms included depression, anxiety, and apathy. Agitation symptoms included disinhibition, agitation, and irritability. Psychotic symptoms included delusions and hallucinations

^b^Model adjusted for baseline variables of age, sex, ethnicity, years of education, APOE e4 status, and use of antidepressants. Significant estimates (with *p* ≤ 0.05) are highlighted in bold

^c^For this outcome measure, the “exchangeable covariance” was used in the mixed linear regression because the model could not converge when the “unstructured covariance” was used

## Discussion

### Summary of findings

This study utilized a large sample and a longitudinal study design to examine the comparative utilities of various NPS in predicting dementia and its subtypes. Three symptom clusters of NPS were identified among cognitively normal older persons, namely psychotic, affective, and agitation symptoms. Although psychotic symptoms were rarely endorsed by the participants, those who did report psychotic symptoms had a relatively higher risk of all-cause dementia compared to those with affective or agitation symptoms. Psychotic symptoms also predicted all the subtypes of dementia, while affective symptoms predicted Alzheimer’s dementia, vascular dementia, and dementia with Lewy Bodies, and agitation symptoms predicted Alzheimer’s dementia, frontotemporal lobar degeneration, and other subtypes of dementia. Across the subtypes of dementia, the risk estimates of psychotic symptoms were relatively higher compared to those of the other two symptom clusters, as well as higher in non-Alzheimer’s dementia compared to Alzheimer’s dementia. Compared to affective and agitation symptoms, psychotic symptoms also predicted a larger magnitude of decline in the *Z*-scores of neuropsychological tests over time, although the 3 symptom clusters demonstrated different profile of decline in the cognitive domains—psychotic symptoms mainly affected the processing speed and executive function, while affective symptoms affected the visuospatial, memory, processing speed, and executive function, and agitation affected language, attention, and executive function.

### Interpretation of findings

Some of the findings from this study are consistent with what have been known in the literature. For example, affective symptoms (such as depression and anxiety) have been shown to predict Alzheimer’s dementia and vascular dementia in recent meta-analyses [6, 7] and have also been included as part of the diagnostic criteria for dementia with Lewy Bodies (as the supportive clinical features for this subtype) [9]. At the same time, this study also added new insight to some of the existing literature. For example, agitation symptoms (such as disinhibition and irritability) were shown to predict Alzheimer’s dementia in this study, which is a less established finding compared to their association with behavioral variant frontotemporal dementia [8]. Similarly, psychotic symptoms were shown to predict all the subtypes of dementia in this study, which is also a less known finding compared to its association with dementia with Lewy Bodies [9].

The finding on the significant association of psychotic symptoms, across all the subtypes of dementia, is probably one of the less expected. Apart from in the context of dementia with Lewy Bodies [9], the association between psychotic symptoms and incident dementia has not been conclusively demonstrated among cognitively normal older persons [43–45]. This is possibly related to the relatively infrequent occurrence of psychotic symptoms in cognitively normal older persons and, consequently, the difficulties in capturing them in research settings. Prior studies that reported the lack of association [46, 47] often had relatively small number of participants with psychotic symptoms, which may limit meaningful interpretation of the results. For example, in one of the studies of 1408 participants [46], there were only 5 participants with delusions and 5 participants with hallucinations, which resulted in a rather wide confidence interval of the reported HR (ranging from 0.08 up to 6.37). On the other hand, prior studies that reported the significant association either did not adjust for key confounders relevant to dementia [48–51] (such as educational attainment and APOE e4 status, and hence may not provide definitive conclusion on the association), or recruited community-dwelling individuals who possibly have had undiagnosed mild cognitive impairment [52–55] (and hence may not be representative of cognitively normal individuals with psychotic symptoms). In contrast to the prior studies, the current study addressed some of the aforementioned limitations in the literature (by capturing a slightly larger number of participants with psychotic symptoms, adjusting for key confounders related to dementia, and including only cognitively normal individuals) and hence may possibly afford a clearer answer on the association between psychotic symptoms and incident dementia among cognitively normal older persons. The finding is also not inconsistent with a recent study of NPS among individuals with mild cognitive impairment [32], which similarly highlighted the higher risk estimates of psychotic symptoms, compared to the other NPS, in predicting incident dementia.

### Potential implications of the findings

As shown in this study, the various NPS in cognitively normal older persons can be useful in predicting dementia and its subtypes, as well as predicting cognitive decline over time. They may aid in identifying high-risk populations, which may then prompt more intensive interventions to prevent cognitive decline (such as those related to risk factor modification, physical exercise, and cognitive training) [56, 57] as well as the consideration of enrolment into preventive trials for dementia. The findings also highlight the need for further research to clarify on the neurobiological links of NPS with cognitive impairment, as well as with the different subtypes of dementia. Prior studies have implicated some of the neurobiological underpinnings of NPS, including the frontal-subcortical circuits and the monoaminergic system in the brain stem [12]. However, the literature remains unclear on whether the various NPS share a common neurobiological pathway, or involve distinct pathways, in the pathogenesis of dementia. The findings from this study provided suggestive evidence that the various NPS potentially involve distinct brain regions (as shown by the differential profile of cognitive decline), which eventually manifest with the different subtypes of dementia. Further research in this area of neurobiological links may potentially enrich our understanding on the pathogenesis of dementia as well as identify novel drug targets to inform the development of disease-modifying drugs for dementia [12].

It is noteworthy that the NPS can remain useful even when they were measured using a screening tool such as the NPI-Q. To date, NPI-Q remains the most commonly used screening tool for NPS in older persons [12, 13] and is one of the few NPS tools that most clinicians in the field are well-versed in. However, NPI-Q was developed primarily to identify NPS in patients with existing cognitive impairment [13] and included the instruction to specifically capture NPS that have occurred “since a patient first began to experience cognitive problems.” Its utility, as well as sensitivity, in capturing NPS among cognitively normal individuals is not yet certain. As shown in this study, even a screening tool such as NPI-Q can be sufficiently useful to capture clinically relevant NPS among cognitively normal individuals. It provides a convenient approach to operationalize the routine profiling of NPS among cognitively normal individuals, which is especially pertinent given the recent inclusion of NPS as being part of the 2018 NIA-AA research framework for Alzheimer’s disease [10]. Notwithstanding the current findings, clinicians who intend to use NPI-Q among cognitively normal individuals may need to adapt the questionnaire, similar to what was done in NACC, to include the following instructions to the NPI-Q interviewer: “For subjects who are cognitively normal or whose cognition has not yet been evaluated, please report any behaviors or symptoms that were present within the last month, ignoring behaviors and symptoms that are usual for the subject and have been customary throughout his/her life.” Future research should also consider further comparative studies between NPI-Q and a separate tool, the Mild Behavioral Impairment–Checklist (MBI-C) [58], which was specifically developed to capture NPS in cognitively normal individuals. NPI-Q and MBI-C have several key differences. Compared to MBI-C, NPI-Q has the strength of being much briefer in length (34 items versus 12 items, respectively). However, MBI-C may be better tailored to individuals without cognitive impairment, with its descriptions on NPS which are more relevant to pre-dementia states. MBI-C also focuses on NPS over a 6-month duration (instead of the 1-month duration in NPI-Q), with an intent to exclude transient NPS such as those related to bereavement, changes in living situations, or transient poor sleep [59]. Future research should investigate whether the differences across these two scales may have any impact on their utilities in dementia prognostication, and whether one may be better than the other in capturing NPS among cognitively normal individuals.

The findings on psychotic symptoms may have implications to the clinical approach in managing psychotic symptoms among cognitively normal older persons. To date, late-life psychotic disorders remain diagnoses of exclusion, often requiring the exclusion of underlying neurodegenerative processes [60, 61]. However, the current diagnostic criteria of late-life psychotic disorders [60, 61] do not dictate the *extent of clinical investigations* that are needed to exclude neurodegenerative processes. This has translated into the prevailing practice where clinicians would not uncommonly exclude dementia based on a cross-sectional assessment (during the initial evaluations) and using cognitive tests which may be insensitive to more subtle cognitive deficits (such as the MMSE). In the presence of apparently *normal cognition* during the *initial evaluations*, the clinical suspicion would often shift to primary psychiatric disorders [60], with the subsequent care focused mostly on relieving psychotic symptoms and less on monitoring for incident dementia [61]. The findings from this study suggest that psychotic symptoms in cognitively normal older persons, albeit being less common than other NPS, may indicate a very high risk of dementia. The apparently *normal cognition* during the *initial evaluations* may not be sufficient to exclude underlying neurodegenerative processes, and there remains a need for continued and close surveillance to detect the onset of cognitive decline in this group of individuals. The findings may have implications to the future diagnostic criteria of late-life psychotic disorders, suggesting the need for greater clarity on the *extent of clinical investigations* in the diagnostic criteria—such as specifying the need for repeated administration of a neuropsychological battery (or in its absence, another briefer but sensitive cognitive test such as the Montreal Cognitive Assessment) [27, 28, 62, 63]—before a diagnosis of late-life psychotic disorder can be accurately made. Potentially, the future diagnostic criteria of late-life psychotic disorders may also incorporate the newer biomarkers of dementia (such as those related to amyloid protein, tau protein, and neuronal injury) [10] to definitively exclude underlying neurodegenerative processes, especially when these biomarkers become more accessible to general clinicians in the foreseeable future. Such approach is not inconsistent with the direction taken by the recently proposed consensus criteria for psychosis in Alzheimer’s disease [64], where there is an increasing emphasis that late-life psychotic disorders may potentially be manifestations of preclinical and prodromal Alzheimer’s disease.

### Limitations

Several limitations should be considered. First, the participants in the study involved those who volunteered at the Alzheimer’s Disease Centers, and may not necessarily represent those in the community. Additional file 8 provides a comparison of the prevalence estimates of NPS between the current sample and a recently published community sample [46]—although the prevalence estimates of NPS in the current study may not be dissimilar from those of the community sample, they are still marginally higher than those from the community sample and are not inconsistent with our understanding that clinical samples may have higher risk of dementia than community samples (and hence also have higher prevalence of NPS than community samples, given that the presence of NPS can be viewed as a marker of higher risk). Notwithstanding the limitation, the findings from this study can be especially relevant to patients in current healthcare services, where patients often present voluntarily to health services (not unlike those who volunteered in this study). Second, this is the first study in the literature to identify the factor structure of NPI-Q among *cognitively normal* older persons. Although the findings provide clear evidence of the three symptom clusters (no cross-loading between the factors in EFA; consistent evidence in CFA; as well as face validity of the symptom clusters), the factor structure of NPI-Q will still benefit from further validation in other populations of cognitively normal older persons. Third, 30.1% of the diagnoses (normal cognition, mild cognitive impairment, or dementia) were made by single clinicians. They may not necessarily be as accurate as those made via consensus conference. Fourth, although this study excluded individuals who reported prior diagnosis or treatment of schizophrenia, there were still a small number of participants (0.7%) who had current use of antipsychotics. It is plausible, though less likely, that this group of participants may have under-reported prior history of schizophrenia and may still be using antipsychotics for the treatment of schizophrenia. However, there is a more plausible explanation to the current use of antipsychotics, where this class of medications is not uncommonly prescribed for adjunct or off-label indications, such as for depressive symptoms, agitation, or even insomnia.

## Conclusion

This study identified three symptom clusters of NPS among cognitively normal older persons and demonstrated their independent yet differential associations with incident dementia. Psychotic symptoms predicted all the subtypes of dementia, while affective and agitation symptoms differentially predicting some subtypes. Psychotic symptoms also had higher risk estimates than affective or agitation symptoms, with its risk estimates being particularly high in non-Alzheimer’s dementia. The findings demonstrate that the various NPS in cognitively normal older persons—even when measured using a screening tool such as NPI-Q—can be useful in predicting dementia and its subtypes, and may potentially aid in identifying high-risk populations for preventive trials and interventions. They highlight the need for further research to clarify the neurobiological links between various NPS and dementia subtypes, which may potentially enrich our understanding on the pathogenesis of neurocognitive disorders. They may also change the clinical approach in managing late-life psychotic symptoms, requiring a greater emphasis on dementia surveillance in the diagnostic criteria of late-life psychotic disorders.

## Supplementary information

**Additional file 1.** Details on the conduct of inverse probability weighting to account for those who dropped out of the study after the first visit.

**Additional file 2.** Comparison of demographic information between participants with and without longitudinal follow-up data.

**Additional file 3 **Scree plot from exploratory factor analysis, based on 80% of the randomly-split sample (*n* = 9962).

**Additional file 4 **Fit indices of various models in confirmatory factor analysis, based on 20% of the randomly-split samples (*n* = 2490).

**Additional file 5.** The first sensitivity analysis using the severity scores of the symptom-clusters.

**Additional file 6.** The second sensitivity analysis to include participants without follow-up data using inverse probability weighting.

**Additional file 7.** The third sensitivity analysis which redefined the symptom-clusters by NPI-Q items with factor loadings of ≥0.20 in the exploratory factor analysis.

**Additional file 8.** Comparison of the prevalence-estimates of neuropsychiatric symptoms between the current sample and those of a previously-published community sample.

## Data Availability

The data were obtained from the National Alzheimer’s Coordinating Center (NACC). For further information on access to the database, please contact NACC (contact details can be found at https://www.alz.washington.edu/WEB/researcher_home.html).

## Abbreviations

CFA: Confirmatory factor analysis
CI: Confidence interval
EFA: Exploratory factor analysis
HR: Hazard ratio
IQR: Interquartile range
IPW: Inverse probability weighting
NACC: National Alzheimer’s Coordinating Center
NIA-AA: National Institute on Aging-Alzheimer’s Association
NPI-Q: Neuropsychiatric Inventory-Questionnaire
NPS: Neuropsychiatric symptoms
USA: United States of America
