# Supplementary material for: Neuropsychiatric symptoms in cognitively normal older persons, and the association with Alzheimer’s and non-Alzheimer’s dementia
Source: Alzheimers Res Ther. 2020 Mar 31;12:35. doi: 10.1186/s13195-020-00604-7 (PMC7110750; doi:10.1186/s13195-020-00604-7)
Supplement: Supplementary file 1 — Additional file 1. Details on the conduct of inverse probability weighting to account for those who dropped out of the study after the first visit. [file 13195_2020_604_MOESM1_ESM.docx]

**Additional file 1.** Details on the conduct of inverse probability weighting to account for those who dropped out of the study after the first visit.

In inverse probability weighting, the “complete cases” (those with follow-up data, n=9,594) were weighted in cox regression by the inverse of their probability of being a complete case so that the results bear more semblance to those who dropped out (n=2,858). The probability of being a complete case was generated from logistic regression, with the predictors based on the variables included in the primary analysis (the three symptom-clusters, age, sex, ethnicity, years of education, APOE e4 status, and use of antidepressants) as well as other auxiliary variables that may help to predict drop-out, including marital status (Married/Widowed/Separated/Single/Other), living arrangement (Alone/Spouse/Relative/Group), type of residence (Private residence/Retirement community/Assisted living), primary reason of participation (Research/Clinical evaluation/Research and clinical evaluation), primary source of referral (Healthcare providers/Non-professional contact/Other), Mini-Mental State Examination score, current smoking, hypertension, hyperlipidemia and diabetes mellitus. This logistic model had an acceptable fit in the Hosmer-Lemeshow test (evidenced by the non-significant p-value of 0.181), with the calibration plot showing agreement between the predicted probability and the observed frequency as shown below:
