# Supplementary material for: Neuropsychiatric symptoms in cognitively normal older persons, and the association with Alzheimer’s and non-Alzheimer’s dementia
Source: Alzheimers Res Ther. 2020 Mar 31;12:35. doi: 10.1186/s13195-020-00604-7 (PMC7110750; doi:10.1186/s13195-020-00604-7)
Supplement: Supplementary file 2 — Additional file 2. Comparison of demographic information between participants with and without longitudinal follow-up data. [file 13195_2020_604_MOESM2_ESM.docx]

**Additional file 2.** Comparison of demographic information between participants with and without longitudinal follow-up data.

| Variable | Overall sample  (n=12,452) | Participants with follow–up data  (n=9,594) | Participants without follow–up data  (n=2,858) | P value ^a^ |
| --- | --- | --- | --- | --- |
| Age, median (IQR) | 72 (67–79) | 73 (67–79) | 71 (66–77) | **<0.001** |
| Male sex, n (%) | 4,514 (36.3) | 3,506 (36.5) | 1,008 (35.3) | 0.210 |
| Ethnicity, n (%) |  |  |  | **<0.001** |
| White | 9,902 (79.5) | 7,719 (80.5) | 2,183 (76.4) |  |
| African American | 1,773 (14.2) | 1,338 (13.9) | 435 (15.2) |  |
| Other / Unknown | 777 (6.2) | 537 (5.6) | 240 (8.4) |  |
| Years of education, median (IQR) | 16 (14–18) | 16 (14–18) | 16 (14–18) | 0.230 |
| Marital status, n (%) |  |  |  | **<0.001** |
| Married | 7,456 (59.9) | 5,775 (60.2) | 1,681 (58.8) |  |
| Widowed | 2,466 (19.8) | 1,975 (20.6) | 491 (17.2) |  |
| Divorced/Separated | 1,745 (14.0) | 1,251 (13.0) | 494 (17.3) |  |
| Single | 685 (5.5) | 519 (5.4) | 166 (5.8) |  |
| Other / Unknown | 100 (0.8) | 74 (0.8) | 26 (0.9) |  |
| Living arrangement, n (%) |  |  |  | **<0.001** |
| Lives alone | 4,105 (33.0) | 3,184 (33.2) | 921 (32.2) |  |
| Lives with spouse | 7,324 (58.8) | 5,666 (59.1) | 1,658 (58.0) |  |
| Lives with relative or friend | 821 (6.6) | 612 (6.4) | 209 (7.3) |  |
| Lives with group / Other | 202 (1.6) | 132 (1.4) | 70 (2.4) |  |
| Type of residence, n (%) |  |  |  | **<0.001** |
| Private residence | 11,251 (90.4) | 8,565 (89.3) | 2,686 (94.0) |  |
| Retirement community | 917 (7.4) | 794 (8.3) | 123 (4.3) |  |
| Assisted living / Nursing home / Other | 284 (2.3) | 235 (2.4) | 49 (1.7) |  |
| Primary reason of participation, n (%) |  |  |  | **<0.001** |
| To participate in research | 10,903 (87.6) | 8,459 (88.2) | 2,444 (85.5) |  |
| For clinical evaluation | 1,240 (10.0) | 959 (10.0) | 281 (9.8) |  |
| For clinical evaluation & participate in research | 298 (2.4) | 166 (1.7) | 132 (4.6) |  |
| Unknown | 11 (0.1) | 10 (0.1) | 1 (0.0) |  |
| Primary source of referral, n (%) |  |  |  | **<0.001** |
| Self/relative/friend | 5,216 (41.9) | 3,924 (40.9) | 1,292 (45.2) |  |
| Healthcare providers | 2,249 (18.1) | 1,557 (16.2) | 692 (24.2) |  |
| Other | 4,584 (36.8) | 3,765 (39.2) | 819 (28.7) |  |
| Unknown | 403 (3.2) | 348 (3.6) | 55 (1.9) |  |
| APOE e4 genotype, n (%) |  |  |  | **<0.001** |
| Two copies of e4 allele | 242 (1.9) | 201 (2.1) | 41 (1.4) |  |
| One copy of e4 allele | 2,528 (20.3) | 2,199 (22.9) | 329 (11.5) |  |
| No e4 allele / Unknown | 9,682 (77.8) | 7,194 (75.0) | 2,488 (87.1) |  |
| Current smoker, n (%) | 589 (4.7) | 464 (4.8) | 125 (4.4) | **0.310** |
| Diabetes mellitus, n (%) | 1,566 (12.6) | 1,146 (11.9) | 420 (14.7) | **<0.001** |
| Hypertension, n (%) | 6,472 (52.0) | 4,987 (52.0) | 1,485 (52.0) | 0.980 |
| Hyperlipidemia, n (%) | 6,449 (51.8) | 4,970 (51.8) | 1,479 (51.7) | 0.960 |
| MMSE score, median (IQR) | 29 (28–30) | 29 (28–30) | 29 (28–30) | 0.250 |
| Use of antidepressants, n (%) | 2,278 (18.3) | 1,686 (17.6) | 592 (20.7) | **<0.001** |
| Presence of NPS, n (%) |  |  |  |  |
| Any NPS | 3,941 (31.6) | 2,923 (30.5) | 1,018 (35.6) | **<0.001** |
| Depression | 1,629 (13.1) | 1,189 (12.4) | 440 (15.4) | **<0.001** |
| Anxiety | 1,107 (8.9) | 798 (8.3) | 309 (10.8) | **<0.001** |
| Apathy | 574 (4.6) | 415 (4.3) | 159 (5.6) | **0.006** |
| Sleep | 1,306 (10.5) | 952 (9.9) | 354 (12.4) | **<0.001** |
| Appetite | 686 (5.5) | 495 (5.2) | 191 (6.7) | **0.002** |
| Agitation | 733 (5.9) | 533 (5.6) | 200 (7.0) | **0.004** |
| Irritability | 1,411 (11.3) | 1,044 (10.9) | 367 (12.8) | **0.004** |
| Disinhibition | 327 (2.6) | 215 (2.2) | 112 (3.9) | **<0.001** |
| Elation | 114 (0.9) | 77 (0.8) | 37 (1.3) | **0.015** |
| Motor disturbance | 160 (1.3) | 99 (1.0) | 61 (2.1) | **<0.001** |
| Delusions | 97 (0.8) | 58 (0.6) | 39 (1.4) | **<0.001** |
| Hallucinations | 41 (0.3) | 24 (0.3) | 17 (0.6) | **0.005** |

IQR, interquartile range; MMSE, Mini-Mental State Examination; NPS, neuropsychiatric symptoms.

^a^ Test of difference between participants with and without longitudinal follow-up data: chi-square test for categorical variables, and Mann-Whitney U test for continuous variables. Bold-faced p-values are ≤0.05.
