# Supplementary material for: Neuropsychiatric symptoms in cognitively normal older persons, and the association with Alzheimer’s and non-Alzheimer’s dementia
Source: Alzheimers Res Ther. 2020 Mar 31;12:35. doi: 10.1186/s13195-020-00604-7 (PMC7110750; doi:10.1186/s13195-020-00604-7)
Supplement: Supplementary file 3 — Additional file 3 Scree plot from exploratory factor analysis, based on 80% of the randomly-split sample (n = 9962). [file 13195_2020_604_MOESM3_ESM.docx]

**Additional file 3.** Scree plot from exploratory factor analysis, based on 80% of the randomly-split sample (n=9,962).

Horn’s parallel analysis indicates the presence of **three factors**, as the first three factors had eigenvalues which were greater than the mean eigenvalues generated from random data matrices. Horn’s parallel analysis^1^ has been recognized as a more accurate method to determine the number of factors in exploratory factor analysis.^2-4^ It is implemented by generating a large number of data matrices from random data and retaining factors that are greater than the mean eigenvalue generated from random data matrices.^2^

**ADDITIONAL REFERENCES**

1. Horn JL. A rationale and test for the number of factors in factor analysis. *Psychometrika.* 1965;30:179-185.

2. Courtney MGR. Determining the Number of Factors to Retain in EFA: Using the SPSS R-Menu v2.0 to Make More Judicious Estimations. Practical Assessment, Research & Evaluation, 18(8). 2015.

3. Gaskin CJ, Happell B. On exploratory factor analysis: a review of recent evidence, an assessment of current practice, and recommendations for future use. *International journal of nursing studies.* 2014;51(3):511-521.

4. Dinno A. Exploring the Sensitivity of Horn’s Parallel Analysis to the Distributional Form of Random Data. *Multivariate behavioral research.* 2009;44(3):362-388.
