# Supplementary material for: Neuropsychiatric symptoms in cognitively normal older persons, and the association with Alzheimer’s and non-Alzheimer’s dementia
Source: Alzheimers Res Ther. 2020 Mar 31;12:35. doi: 10.1186/s13195-020-00604-7 (PMC7110750; doi:10.1186/s13195-020-00604-7)
Supplement: Supplementary file 4 — Additional file 4 Fit indices of various models in confirmatory factor analysis, based on 20% of the randomly-split samples (n = 2490). [file 13195_2020_604_MOESM4_ESM.docx]

**Additional file 4.** Fit indices of various models in confirmatory factor analysis, based on 20% of the randomly-split samples (n=2,490). The model that fulfilled the criteria of excellent fit is highlighted in bold.

| CFA model | RMSEA^a^ | SRMR^a^ | CFI^a^ | TLI^a^ |
| --- | --- | --- | --- | --- |
| One-factor model  (Unidimentional) ^b^ | 0.059 | 0.054 | 0.87 | 0.84 |
| **Three-factor model from EFA of this study**  **(Affective, Agitation, Psychotic) ^c^** | **0.030** | **0.031** | **0.98** | **0.97** |
| Three-factor model from EFA of this study, based on factor loading ≥0.20  (Affective, Agitation, Psychotic) ^d^ | 0.051 | 0.049 | 0.91 | 0.88 |
| Four-factor model  (Affective, Agitation, Psychotic, Sleep/Appetite/Elation/Motor disturbance) ^e^ | 0.051 | 0.045 | 0.91 | 0.88 |
| Five-factor model  (Affective, Agitation, Psychotic, Sleep/Appetite, Elation/Motor disturbance) ^f^ | 0.050 | 0.043 | 0.92 | 0.88 |

CFA, confirmatory factor analysis; EFA, exploratory factor analysis; RMSEA, root mean square error of approximation; SRMR, standardized root mean square residual; CFI, comparative fit index; TLI, Tucker-Lewis index.

^a^ A model is considered to have excellent fit if it fulfils all of the following four criteria: RMSEA≤0.05, SRMR≤0.05, CFI≥0.95, and TLI≥0.95.

^b^ The one-factor model indicates Neuropsychiatric Inventory-Questionnaire as a unidimensional scale.

^c^ This three-factor model comprises Affective symptoms (*depresion*, *anxiety* and *apathy*), Agitation symptoms (*disinhibition*, *agitation* and *irritability*), and Psychotic symptoms (*delusions* and *hallucinations*).

^d^ This three-factor model includes items that loaded in EFA (based on factor loading of ≥0.40), as well as those with marginal factor loading of 0.20-0.40 (*sleep*, *appetite*,  *elation*, and *motor disturbance*). It comprises Affective symptoms (*depresion*, *anxiety*, *apathy, sleep* and *appetite*), Agitation symptoms (*disinhibition*, *agitation*, *irritability*, *elation* and *motor disturbance*), and Psychotic symptoms (*delusions* and *hallucinations*).

^e^ This four-factor model includes the three factors in EFA (based on factor loading of ≥0.40), as well as an additional factor for items with marginal factor loading of 0.20-0.40 (*sleep*, *appetite*,  *elation*, and *motor disturbance*). Specifically, it comprises Affective symptoms (*depresion*, *anxiety* and *apathy*), Agitation symptoms (*disinhibition*, *agitation* and *irritability*), Psychotic symptoms (*delusions* and *hallucinations*), and an additional factor (*sleep*, *appetite*,  *elation*, and *motor disturbance*).

^f^ This five-factor model includes the three factors in EFA (based on factor loading of ≥0.40), as well as two additional factors for items with marginal factor loading of 0.20-0.40 (*sleep*, *appetite*,  *elation*, and *motor disturbance*). Specifically, it comprises Affective symptoms (*depresion*, *anxiety* and *apathy*), Agitation symptoms (*disinhibition*, *agitation* and *irritability*), Psychotic symptoms (*delusions* and *hallucinations*), a separate factor for *sleep* and *appetite*, as well as a separate factor for *elation* and *motor disturbance*.
