# Supplementary material for: Neuropsychiatric symptoms in cognitively normal older persons, and the association with Alzheimer’s and non-Alzheimer’s dementia
Source: Alzheimers Res Ther. 2020 Mar 31;12:35. doi: 10.1186/s13195-020-00604-7 (PMC7110750; doi:10.1186/s13195-020-00604-7)
Supplement: Supplementary file 5 — Additional file 5. The first sensitivity analysis using the severity scores of the symptom-clusters. [file 13195_2020_604_MOESM5_ESM.docx]

**Additional file 5.** The first sensitivity analysis using the severity scores of the symptom-clusters.

| Dementia aetiology | Affective symptoms  (severity score) ^a^ | |  | Agitation symptoms  (severity score) ^a^ | | |  | | Psychotic symptoms  (severity score) ^a^ | | |
| --- | --- | --- | --- | --- | --- | --- | --- | --- | --- | --- | --- |
|  | HR (95% CI) ^b, c^ | P–value |  | HR (95% CI) ^b, c^ | P–value |  | | HR (95% CI) ^b, c^ | | P–value |  |
| All–cause dementia | **1.2 (1.1–1.3)** | **<0.001** |  | **1.2 (1.1–1.3)** | **<0.001** |  | | **1.7 (1.3–2.3)** | | **<0.001** |  |
| Alzheimer’s dementia | **1.1 (1.0–1.3)** | **0.006** |  | **1.2 (1.1–1.3)** | **<0.001** |  | | **1.5 (1.0–2.0)** | | **0.025** |  |
| Vascular dementia | **1.3 (1.1–1.7)** | **0.016** |  | 1.1 (0.8–1.4) | 0.730 |  | | **1.9 (1.2–2.9)** | | **0.004** |  |
| Dementia with Lewy Bodies | **1.4 (1.2–1.8)** | **0.001** |  | 1.0 (0.7–1.4) | 0.951 |  | | **2.1 (1.3–3.4)** | | **0.002** |  |
| Frontotemporal lobar degeneration | 1.2 (0.8–1.7) | 0.341 |  | **2.0 (1.4–2.8)** | **<0.001** |  | | **2.0 (1.2–3.4)** | | **0.008** |  |
| Other or unknown subtypes of dementia | 1.0 (0.7–1.3) | 0.827 |  | **1.4 (1.1–1.8)** | **0.005** |  | | **1.6 (1.0–2.4)** | | **0.032** |  |

HR, hazard ratio.

^a^ Affective symptoms included depression, anxiety and apathy. Agitation symptoms included disinhibition, agitation and irritability. Psychotic symptoms included delusions and hallucinations.

^b^ Model adjusted for baseline variables of age, sex, ethnicity, years of education, APOE e4 status, and use of antidepressants. Significant risk-estimates (with p≤0.05) are highlighted in bold.

^c^ The hazard ratios were based on each unit increase in the severity scores of the symptom-clusters.
