# Supplementary material for: Neuropsychiatric symptoms in cognitively normal older persons, and the association with Alzheimer’s and non-Alzheimer’s dementia
Source: Alzheimers Res Ther. 2020 Mar 31;12:35. doi: 10.1186/s13195-020-00604-7 (PMC7110750; doi:10.1186/s13195-020-00604-7)
Supplement: Supplementary file 6 — Additional file 6. The second sensitivity analysis to include participants without follow-up data using inverse probability weighting. [file 13195_2020_604_MOESM6_ESM.docx]

**Additional file 6.** The second sensitivity analysis to include participants without follow-up data using inverse probability weighting.

| Dementia aetiology | Presence of Affective symptoms ^a^ | |  | Presence of Agitation symptoms ^a^ | | |  | | Presence of Psychotic symptoms ^a^ | |
| --- | --- | --- | --- | --- | --- | --- | --- | --- | --- | --- |
|  | HR (95% CI) ^b^ | P–value |  | HR (95% CI) ^b^ | P–value |  | | HR (95% CI) ^b^ | | P–value |
| All–cause dementia | **1.5 (1.2–1.8)** | **<0.001** |  | **1.6 (1.3–2.1)** | **<0.001** |  | | **3.8 (2.2–6.8)** | | **<0.001** |
| Alzheimer’s dementia | **1.4 (1.1–1.7)** | **0.018** |  | **1.7 (1.3–2.2)** | **<0.001** |  | | **2.3 (1.2–4.6)** | | **0.018** |
| Vascular dementia | **2.0 (1.1–3.7)** | **0.030** |  | 1.2 (0.6–2.5) | 0.577 |  | | **6.0 (1.3–27.0)** | | **0.019** |
| Dementia with Lewy Bodies | **2.8 (1.5–5.2)** | **0.001** |  | 0.7 (0.3–1.8) | 0.461 |  | | **16.6 (4.6–59.9)** | | **<0.001** |
| Frontotemporal lobar degeneration | 2.6 (0.8–9.3) | 0.129 |  | **4.1 (1.2–13.9)** | **0.026** |  | | **9.6 (2.5–36.7)** | | **0.001** |
| Other or unknown subtypes of dementia | 1.2 (0.6–2.5) | 0.546 |  | **2.3 (1.1–4.5)** | **0.020** |  | | **4.7 (1.5–14.5)** | | **0.008** |

NPI-Q, Neuropsychiatric Inventory–Questionnaire; HR, hazard ratio.

^a^ Affective symptoms included depression, anxiety and apathy. Agitation symptoms included disinhibition, agitation and irritability. Psychotic symptoms included delusions and hallucinations.

^b^ Model adjusted for baseline variables of age, sex, ethnicity, years of education, APOE e4 status, and use of antidepressants. Significant risk-estimates (with p≤0.05) are highlighted in bold.
