# Supplementary material for: Neuropsychiatric symptoms in cognitively normal older persons, and the association with Alzheimer’s and non-Alzheimer’s dementia
Source: Alzheimers Res Ther. 2020 Mar 31;12:35. doi: 10.1186/s13195-020-00604-7 (PMC7110750; doi:10.1186/s13195-020-00604-7)
Supplement: Supplementary file 7 — Additional file 7. The third sensitivity analysis which redefined the symptom-clusters by NPI-Q items with factor loadings of ≥0.20 in the exploratory factor analysis. [file 13195_2020_604_MOESM7_ESM.docx]

**Additional file 7.** The third sensitivity analysis which redefined the symptom-clusters by NPI-Q items with factor loadings of ≥0.20 in the exploratory factor analysis.

| Dementia aetiology | Presence of Affective symptoms ^a^ | |  | Presence of Agitation symptoms ^a^ | | |  | | Presence of Psychotic symptoms ^a^ | |
| --- | --- | --- | --- | --- | --- | --- | --- | --- | --- | --- |
|  | HR (95% CI) ^b^ | P–value |  | HR (95% CI) ^b^ | P–value |  | | HR (95% CI) ^b^ | | P–value |
| All–cause dementia | **1.7 (1.4-2.0)** | **<0.001** |  | **1.5 (1.2-1.8)** | **0.002** |  | | **3.7 (2.1-6.5)** | | **<0.001** |
| Alzheimer’s dementia | **1.5 (1.2-1.9)** | **<0.001** |  | **1.5 (1.2-2.0)** | **0.003** |  | | **2.3 (1.1-4.6)** | | **0.025** |
| Vascular dementia | **2.1 (1.2-3.8)** | **0.010** |  | 1.0 (0.5-2.1) | 0.959 |  | | **6.3 (1.4-27.9)** | | **0.016** |
| Dementia with Lewy Bodies | **2.0 (1.0-3.8)** | **0.040** |  | 0.8 (0.3-1.9) | 0.625 |  | | **14.5 (4.0-53.0)** | | **<0.001** |
| Frontotemporal lobar degeneration | **4.0 (1.3-12.2)** | **0.016** |  | **3.4 (1.3-9.0)** | **0.012** |  | | **8.8 (1.9-41.2)** | | **0.005** |
| Other or unknown subtypes of dementia | 1.5 (0.8-2.9) | 0.231 |  | **2.3 (1.2-4.5)** | **0.018** |  | | **4.8 (1.6-14.7)** | | **0.005** |

NPI-Q, Neuropsychiatric Inventory–Questionnaire; HR, hazard ratio.

^a^ Affective symptoms included depression, anxiety, apathy, sleep and appetite. Agitation symptoms included disinhibition, agitation, irritability, elation and motor disturbance. Psychotic symptoms included delusions and hallucinations.

^b^ Model adjusted for baseline variables of age, sex, ethnicity, years of education, APOE e4 status, and use of antidepressants. Significant risk-estimates (with p≤0.05) are highlighted in bold.
