# Supplementary material for: Neuropsychiatric symptoms in cognitively normal older persons, and the association with Alzheimer’s and non-Alzheimer’s dementia
Source: Alzheimers Res Ther. 2020 Mar 31;12:35. doi: 10.1186/s13195-020-00604-7 (PMC7110750; doi:10.1186/s13195-020-00604-7)
Supplement: Supplementary file 8 — Additional file 8. Comparison of the prevalence-estimates of neuropsychiatric symptoms between the current sample and those of a previously-published community sample. [file 13195_2020_604_MOESM8_ESM.docx]

**Additional file 8.**  Comparison of the prevalence-estminates of neuropsychiatric symptoms between the current sample and those of a previously-published community sample.^1^

| Neuropsychiatric symptoms | Current sample  (n=12,452) | A previously-published community sample  (n=1,408) ^1^ |
| --- | --- | --- |
| Depression | 1,629 (13.1%) | 153 (10.9%) |
| Anxiety | 1,107 (8.9%) | 66 (4.7%) |
| Apathy | 574 (4.6%) | 57 (4.0%) |
| Sleep | 1,306 (10.5%) | 122 (10.7%) ^a^ |
| Appetite | 686 (5.5%) | 67 (4.8%) |
| Agitation | 733 (5.9%) | 33 (2.3%) |
| Irritability | 1,411 (11.3%) | 96 (6.8%) |
| Disinhibition | 327 (2.6%) | 22 (1.6%) |
| Elation | 114 (0.9%) | 7 (0.5%) |
| Motor disturbance | 160 (1.3%) | 7 (0.5%) |
| Delusions | 97 (0.8%) | 5 (0.4%) |
| Hallucinations | 41 (0.3%) | 5 (0.4%) |

^a^ The percentage was calculated based on a sample size of 1,137 (as 271 participants did not provide data on sleep).

**ADDITIONAL REFERENCES**

1. Geda YE, Roberts RO, Mielke MM, et al. Baseline neuropsychiatric symptoms and the risk of incident mild cognitive impairment: a population-based study. *The American journal of psychiatry.* 2014;171(5):572-581.
